# Low HIF-1α and low EGFR mRNA Expression Significantly Associate with Poor Survival in Soft Tissue Sarcoma Patients; the Proteins React Differently

**DOI:** 10.3390/ijms19123842

**Published:** 2018-12-03

**Authors:** Swetlana Rot, Helge Taubert, Matthias Bache, Thomas Greither, Peter Würl, Hans-Jürgen Holzhausen, Alexander W. Eckert, Dirk Vordermark, Matthias Kappler

**Affiliations:** 1 Department of Oral and Maxillofacial Plastic Surgery, Martin Luther University Halle-Wittenberg, 06120 Halle (Saale), Germany; swetlana.rot@medizin.uni-halle.de (S.R.); alexander.eckert@uk-halle.de (A.W.E.); 2 Clinic of Urology and Pediatric Urology, FA University Hospital Erlangen-Nuremberg, 91054 Erlangen, Germany; Helge.Taubert@uk-erlangen.de; 3 Department of Radiotherapy, Martin Luther University Halle-Wittenberg, 06120 Halle (Saale), Germany; matthias.bache@medizin.uni-halle.de (M.B.); dirk.vordermark@uk-halle.de (D.V.); 4 Center for Reproductive Medicine and Andrology, Martin Luther University, 06120 Halle (Saale), Germany; thomas.greither@medizin.uni-halle.de; 5 Department of General and Visceral Surgery, Hospital Dessau, 06847 Dessau-Roßlau, Germany; peter.wuerl@klinikum-dessau.de; 6 Institute of Pathology, Martin Luther University Halle-Wittenberg, 06120 Halle (Saale), Germany; Hans-juergen.holzhausen@medizin.uni-halle.de

**Keywords:** *EGFR*, HIF1α, STS, prognosis

## Abstract

In various tumors, the hypoxia inducible factor-1α (*HIF-1α*) and the epidermal growth factor-receptor (*EGFR*) have an impact on survival. Nevertheless, the prognostic impact of both markers for soft tissue sarcoma (STS) is not well studied. We examined 114 frozen tumor samples from adult soft tissue sarcoma patients and 19 frozen normal tissue samples. The mRNA levels of HIF-1α, EGFR, and the reference gene hypoxanthine phosphoribosyltransferase (HPRT) were quantified using a multiplex qPCR technique. In addition, levels of EGFR or HIF-1α protein were determined from 74 corresponding protein samples using ELISA techniques. Our analysis showed that a low level of HIF-1α or EGFR mRNA (respectively, relative risk (RR) = 2.8; *p* = 0.001 and RR = 1.9; *p* = 0.04; multivariate Cox´s regression analysis) is significantly associated with a poor prognosis in STS patients. The combination of both mRNAs in a multivariate Cox’s regression analysis resulted in an increased risk of early tumor-specific death of patients (RR = 3.1, *p* = 0.003) when both mRNA levels in the tumors were low. The EGFR protein level had no association with the survival of the patient’s cohort studied, and a higher level of HIF-1α protein associated only with a trend to significance (multivariate Cox’s regression analysis) to a poor prognosis in STS patients (RR = 1.9, *p* = 0.09). However, patients with low levels of HIF-1α protein and a high content of EGFR protein in the tumor had a three-fold better survival compared to patients without such constellation regarding the protein level of HIF-1α and EGFR. In a bivariate two-sided Spearman’s rank correlation, a significant correlation between the expression of HIF-1α mRNA and expression of EGFR mRNA (*p* < 0.001) or EGFR protein (*p* = 0.001) was found, additionally, EGFR mRNA correlated with EGFR protein level (*p* < 0.001). Our results show that low levels of HIF-1α mRNA or EGFR mRNA are negative independent prognostic markers for STS patients, especially after combination of both parameters. The protein levels showed a different effect on the prognosis. In addition, our analysis suggests a possible association between HIF-1α and EGFR expression in STS.

## 1. Background

Soft tissue sarcomas (STS) are a heterogeneous group of relatively aggressive tumors of mesenchymal origin [1,2]. The treatment options for STS are often limited to surgery with consideration for chemotherapy and radiotherapy [2]. Therefore, new prognostic markers are required that have the potential to assess the effectiveness of an individual therapeutic strategy.

A potential prognostic marker for STS could be hypoxia inducible factor-1α (*HIF-1α*), which is the inducible subunit of the transcription factor HIF-1. It is commonly thought to be the most important marker of mammalian cell transcriptional response to oxygen deprivation. HIF-1α plays an important role in the formation of solid tumors by promoting angiogenesis and anaerobic metabolism [3]. High expression of HIF-1α protein in various cancers including cervical, head and neck and oropharyngeal carcinoma was correlated with an unfavorable prognosis [4,5,6]. However, in other studies, high expression of HIF-1α protein correlated with good prognosis, such as lung cancer, squamous cell carcinoma, or renal cell carcinoma [7,8,9].

To the best of our knowledge, up to now, three immunohistochemical studies examined the prognostic impact of HIF-1α protein in STS. Kim et al., 2015 identified HIF-1α protein as an independent prognostic factor in 55 samples of STS [10]. Forker et al., 2018 did not find such an association in a multivariate Cox’s regression analysis, but they found HIF-1α protein to be prognostically relevant in a univariate analysis [11]. However, Smeland et al., found no association between the detection of HIF-1α protein and the prognosis of STS patients [12]. Other authors already suggested that HIF-1α is not important for tumor formation, but has a significant impact on sarcoma metastasis [13]. The overexpression of hypoxia-induced genes like *HIF-1α* in metastasizing primary tumors provides a basis for further studies of hypoxia in STS to clarify its role in metastasis [14]. This view regarding the influence of HIF’s on the metastasis processes could be an already accepted statement for this marker, based on the work by Rankin and Gaccia [15].

It is known that HIF-1α protein expression, especially when occurring in a diffuse pattern, is not always associated with hypoxia. Often, a diffuse HIF-1α protein expression pattern can be attributed to changes in the expression of oncogenes or tumor suppressor genes [3]. Shintani et al. found that HIF-1α protein had a diffuse staining in STS-samples. Therefore, these authors suggested that HIF-1α protein expression is regulated by various non-oxygen-dependent mechanisms such as the *PI3K* and *MAPK* pathways in STS [16]. Such a statement was also made by other author for breast cancer cells [17].

The multiple-described relationship of HIF-1α and the epidermal growth factor-receptor (*EGFR)/pAKT* pathway also makes this link interesting for STS [17,18], because epidermal growth factor can initiate an intracellular signal transduction cascade involving the *PI3K/AKT* and *MAPK* signaling pathways. Finally, overexpression of EGFR protein was also demonstrated for soft tissue sarcoma [19,20]. However, the effect of EGFR on the metastasis status of STS patients is controversially discussed [14,21,22].

In this study, we analyzed the prognostic effects of EGFR and HIF-1α mRNA/protein expression and their in vivo interaction. We were able to show that low EGFR mRNA expression and low HIF-1α mRNA expression are associated with a poorer prognosis for STS patients, but in particular, the combination of both markers appears to be a helpful tool to assess the risk of tumor-related death of STS patients.

## 2. Results

### 2.1. Expression Level of EGFR- and HIF-1α mRNA in STS

The median transcript ratios of the 114 STS samples examined were 4.7 copies of HIF-1α mRNA per hypoxanthine phosphoribosyltransferase (HPRT) mRNA copy (ranging from 0.3–109.9; mean 10.0) and 1.5 copies EGFR mRNA per HPRT mRNA copy (ranging from 0–161.7; mean 7.1). In contrast, the median transcript ratios of the 19 adjacent non-tumor tissues were 0.2 copies HIF-1α mRNA per HPRT mRNA copy (ranging from 0–1.6; mean 0.3) and 0.06 copies EGFR mRNA per HPRT mRNA copy (range 0–0.3, mean 0.07). For survival analysis, we separated the cohort of STS patients into two groups according to the median expression of HIF-1α and EGFR mRNA. High-level expression of HIF-1α and EGFR was defined as a relative value above 4.7 copies HIF1α mRNA and 1.5 copies EGFR mRNA per HPRT mRNA copy, respectively (Table 1).

### 2.2. Effect of the Expression Level of EGFR- and HIF-1α mRNA on Survival

**Figure 1:**
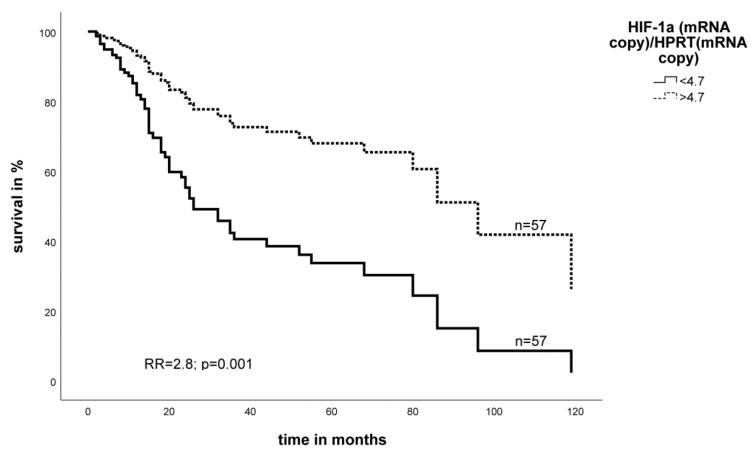
Multivariate Cox’s hazard regression model for HIF-1α mRNA expression and tumor-specific survival in STS patients. The expression of HIF-1α mRNA for 114 STS patients was associated with survival. The model was adjusted for tumor stage, tumor localization, tumor entity, and the type of tumor resection. The high and low cut-off values for HIF-1α were >4.7 and ≤4.7 copies of HIF-1α mRNA relative to the number of copies of hypoxanthine phosphoribosyltransferase (HPRT) mRNA, respectively (relative risk (RR) = 2.8, *p* = 0.001).

**Figure 2:**
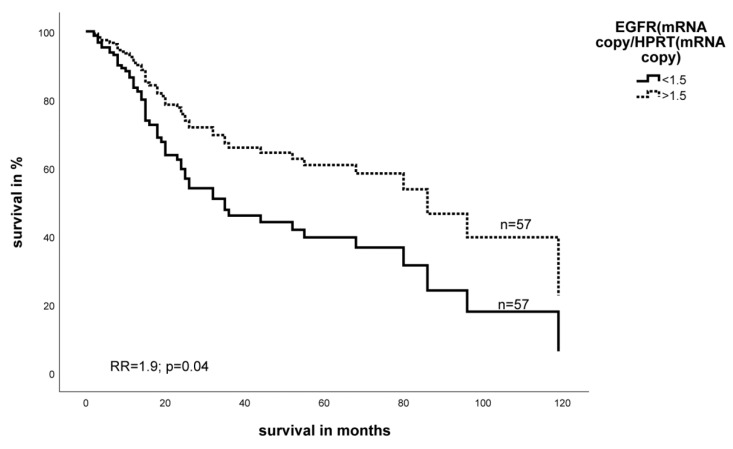
Multivariate Cox’s hazard regression model for EGFR mRNA expression and tumor-specific survival in STS patients.Expression of EGFR for 114 STS patients was associated with survival. The model was adjusted for tumor stage, tumor localization, tumor entity, and the type of tumor resection. The high and low cut-off values for EGFR were >1.5 and ≤1.5 copies of EGFR mRNA relative to the number of copies of HPRT mRNA, respectively (RR = 1.9, *p* = 0.04).

We performed a multivariate Cox’s proportional hazards analysis adjusted for tumor stage, tumor entity, tumor localization, and type of tumor resection. We found that a low level of HIF-1α mRNA is significantly associated with a 2.8-fold increased risk of tumor-related death in STS patients (*p* = 0.001) (Figure 1). In addition, multivariate Cox’s regression analysis demonstrated that low EGFR mRNA levels also associate significantly with poor prognosis in STS patients (RR = 1.9, *p* = 0.04) (Figure 2).

**Figure 3:**
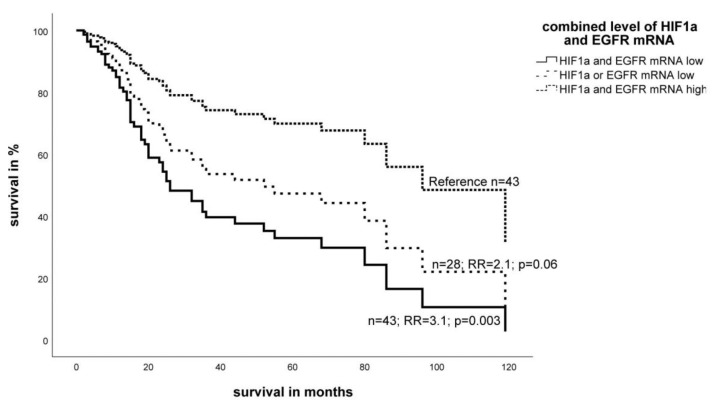
Multivariate Cox’s hazard regression model for the combination of HIF-1α and EGFR mRNA expression and tumor-specific survival in STS patients. The expression of HIF-1α mRNA and EGFR mRNA for 114 STS patients was associated with survival. The model was adjusted for tumor stage, tumor localization, tumor entity, and the type of tumor resection. If both markers had a low expression in the tumor, the prognosis of the patients was worse (RR = 3.1, *p* = 0.003) compared to high expression of both markers in the tumor.

To determine the common effect of HIF-1α mRNA and EGFR mRNA, three groups were defined according to the expression of both markers: (I) high expression of both markers, (II) high expression of only one marker, and (III) low expression of both markers in the tumor. In the multivariate Cox’s regression analysis, low expression of both markers in STS patients resulted in a significantly worse prognosis (RR = 3.1, *p* = 0.003) compared to high expression of both markers in the tumor (Figure 3). This effect was stronger than those from just one marker.

Considering only patients with no recurrence after the primary tumor resection, HIF-1α mRNA (RR = 0.8, *p* = 0.57) (data not shown) levels had no impact on survival in the univariate Cox’s regression hazard analysis. However, those patients with recurrence after the primary tumor resection had a 3.1-fold (univariate Cox’s regression hazard analysis) increased risk of tumor-related death when the expression was less than 4.7 copies of HIF-1α mRNA per copy of HPRT mRNA in their primary tumors (*p* = 0.003). Patients with high HIF-1α mRNA expression had a recurrence after a mean of 19 months; whereas patients with a low HIF-1α mRNA expression had a recurrence after a mean of 14 months after the diagnosis of the primary tumor. However, such an association was not found when analyzing the expression of EGFR-mRNA in STS patients (data not shown).

### 2.3. Expression Level of EGFR- and HIF-1α Protein in STS and Impact on Survival

The median ratios of the 74 STS protein samples were 55 pg HIF-1α per µg protein in the samples (ranging from 0–2545; mean 131) and 0.76 ng EGFR protein per µg protein of the samples (ranging from 0–22.8; mean 2.4). For the subsequent survival analysis, we separated the cohort of STS patients into two equal groups according to the median expression of HIF-1α or EGFR protein level.

**Figure 4:**
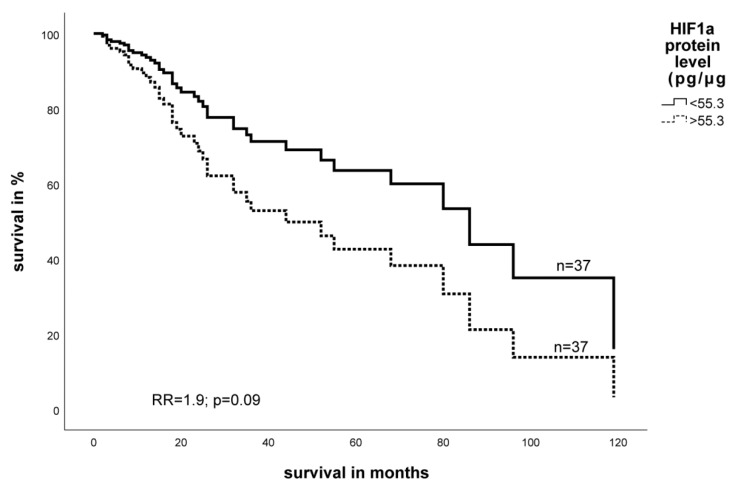
Multivariate Cox’s hazard regression model for HIF-1α protein expression and tumor-specific survival in STS patients. The expression of HIF-1α protein for 74 STS patients was associated with survival. The model was adjusted for tumor stage, tumor localization, tumor entity, and the type of tumor resection. The high and low cut-off values for HIF-1α were >55 and ≤55 pg HIF-1α per µg protein, respectively (RR = 1.9, *p* = 0.09).

**Figure 5:**
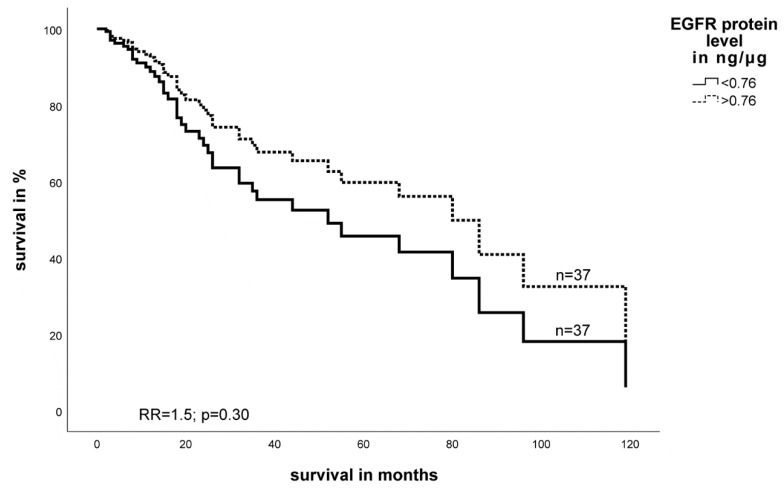
Multivariate Cox’s hazard regression model for EGFR protein expression and tumor-specific survival in STS patients. Expression of EGFR protein for 74 STS patients was associated with survival. The model was adjusted for tumor stage, tumor localization, tumor entity, and the type of tumor resection. The high and low cut-off values for EGFR were >0.76 and ≤0.76 ng EGFR protein per µg protein, respectively (RR = 1.5, *p* = 0.30).

The multivariate Cox’s proportional hazards analysis regression model (adjusted for tumor stage, tumor entity, tumor localization, and type of tumor resection) revealed for a higher HIF-1α protein level a trend towards significance for a poor prognosis in STS patients (RR = 1.9; *p* = 0.09) (Figure 4). For the EGFR protein level, there was no association with the tumor-specific survival of the examined patient’s cohort, However, lower levels of EGFR protein, similar to the RNA analysis of this marker, were somewhat associated with a poor survival of patients (RR = 1.5, *p* = 0.30) (Figure 5). 

**Figure 6:**
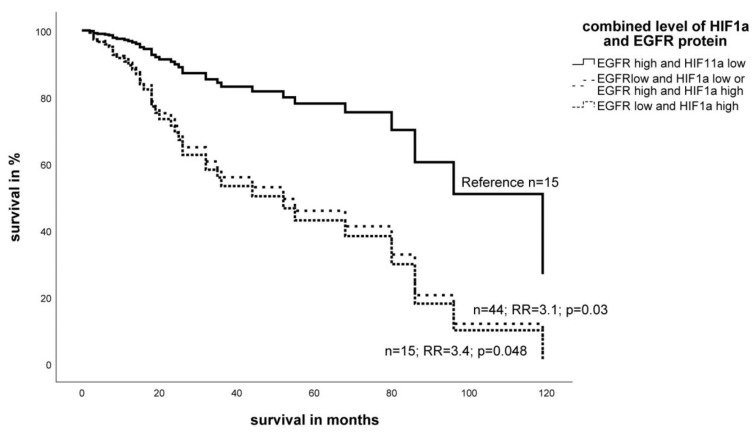
Multivariate Cox’s hazard regression model for the combination of HIF-1α and EGFR protein expression and tumor-specific survival in STS patients. The expression of HIF-1α protein and EGFR protein for 74 STS patients was associated with survival. The model was adjusted for tumor stage, tumor localization, tumor entity, and the type of tumor resection. A low HIF-1α protein and a high EGFR protein level in the tumor resulted in a significantly better prognosis in STS patients compared to that of groups II and III.

Similar to the measurements of mRNA levels, we also combined the expression of HIF-1α protein and EGFR-protein levels for survival analysis. Since higher HIF-1α protein levels and lower EGFR protein levels were associated with worse prognosis, we decided to define the following three groups for the protein analysis: (I) high HIF-1α protein and a low EGFR protein levels, (II) both markers with high or both with low expression, and (III) low HIF-1α protein and a high EGFR protein level. In a multivariate Cox’s regression analysis, a low HIF-1α protein and a high EGFR protein level in the tumor were significantly associated with a better prognosis in STS patients compared to the groups II and III that showed similarly poor prognosis (RR = 3.1; *p* = 0.03 and RR = 3.4; *p* = 0.048; Figure 6).

### 2.4. Correlation of Biomarker Expression and Clinico-Pathological Data

A bivariate two-sided Spearman’s rank correlation showed a significant correlation between HIF-1α mRNA and EGFR mRNA expression in tumor tissues (correlation coefficient: +0.69; *p* < 0.001). A significant correlation between HIF-1α mRNA and EGFR mRNA expression was also found in the associated normal tissues (correlation coefficient: +0.46; *p* = 0.047).

In tumor tissue, the HIF-1α mRNA level correlated with the EGFR protein level (correlation coefficient: +0.36; *p* = 0.001), in addition, the EGFR mRNA correlated with EGFR protein level (correlation coefficient: +0.53; *p* < 0.001). By means of a Kruskal Wallis test, EGFR-protein expression was also associated with the tumor type (*p* = 0.04), see Table 2. However, an association of EGFR or HIF-1α to the lymph node status was not found. 

## 3. Discussion

In this study, we found by multivariate Cox’s regression analysis that both low EGFR and low HIF-1α mRNA levels are independent negative prognostic markers for tumor-specific survival in analyzed STS patients. The prognostic effect was further increased by combining both mRNAs of EGFR and HIF-1α in comparison to a low expression of a single marker. Furthermore, patients, who had a recurrence with primary tumors exhibiting low levels of HIF-1α mRNA have a poorer prognosis compared to patients with primary tumors exhibiting high levels of HIF-1α mRNA (data not shown). Although the protein level of any single marker did not have a significant effect on survival of patients, the combination of both protein markers demonstrated an impact on the prognosis for STS patients.

There are only a few studies evaluating the relationship between HIF-1α mRNA expression and the outcome of soft tissue sarcoma patients [23]. In a previous study on a subgroup of 45 patients, of the 114 STS samples we studied, an association between low HIF-1α mRNA expression and an unfavorable outcome was found [23]. However, a study in esophageal squamous cell carcinomas found no association between prognosis and HIF-1α mRNA expression [24].

We did not find a correlation between HIF-1α mRNA and protein levels. However, some studies described a linear correlation between the HIF-1α mRNA and HIF-1α protein expression for several types of cancers [25,26]. A high expression of HIF-1α mRNA and protein was accompanied by an increase in the copy number of *HIF-1α* DNA in HNSCC [26]. However, the prognostic impact of high HIF-1α protein expression is controversially discussed [4,6,8,9]. In a study of 49 STS patients, Shintani et al. reported a significant independent association of HIF-1α protein overexpression detected by semiquantitative immunohistochemistry with an unfavorable prognosis [16].

The postulated correlation between HIF-1α and EGFR is still under debate. A relationship between HIF-1α and EGFR has been described by Semenza [3]. He reports that growth factors such as insulin-like growth factor-2 (*IGF2*) and transforming growth factor-α (*TGF-α*) are also HIF-1 target genes. Binding of these factors to their receptors, i.e., insulin-like growth factor 1 receptor (*IGF1R*) and epidermal growth-factor receptor (*EGFR*), respectively, can activate signal-transduction pathways that lead again to HIF-1α expression. Peng et al. suggest that HIF-1α detection might be caused by a normoxic activation of the *PI3K* pathway after EGFR activation [17]. On the other hand, inhibition of HIF-1α by galbanic acid can result in a shortened half-life and degradation of EGFR protein [27].

Expression patterns of EGFR have already been described for STS by immunohistochemical analyses [19,28,29,30]. However, these studies did not show a significant correlation between EGFR expression and prognosis in STS patients. To the best of our knowledge, this is the first study to find an association of low EGFR mRNA expression with tumor-specific survival in STS patients. An association of detection of EGFR positive STS cells with metastasis has been demonstrated [21]. However, we did not find such an association of the mRNA or protein level of EGFR or HIF-1α with the metastasis status of the investigated STS cohort.

Yang et al. found that the activated, phosphorylated form of EGFR (pEGFR) is a prognostic factor for STS patients, but not the total EGFR level [31]. EGFR mRNA expression has been already investigated in other STS cell lines, however, an effect of EGFR on the migration and proliferation in the investigated myxofibrosarcoma cell line was not found [32]. These findings could be of therapeutic importance since the activated form of EGFR is also in the focus of anti-EGFR therapeutic approaches for STS patients [33].

In a previous study analyzing the same STS cohort, we showed that a lower ERBB2 (*HER2/NEU*) mRNA level correlates with a poorer prognosis in STS patients (RR = 3.0; *p* < 0.001) [34]. The results of the current study are in line with that previous data in that low mRNA levels of EGFR or HIF-1α mRNA are associated with poor tumor-specific survival.

In a univariate survival analysis for non-small cell lung cancer, a high EGFR mRNA expression was significantly associated with a prolonged progression-free survival. Furthermore, high EGFR mRNA expression correlated with an increased *EGFR* gene copy number [35]. In addition, overexpression of EGFR mRNA was significantly associated with a worse prognosis in astrocytoma, breast, and gastric cancer patients [36,37,38].

**Figure 7:**
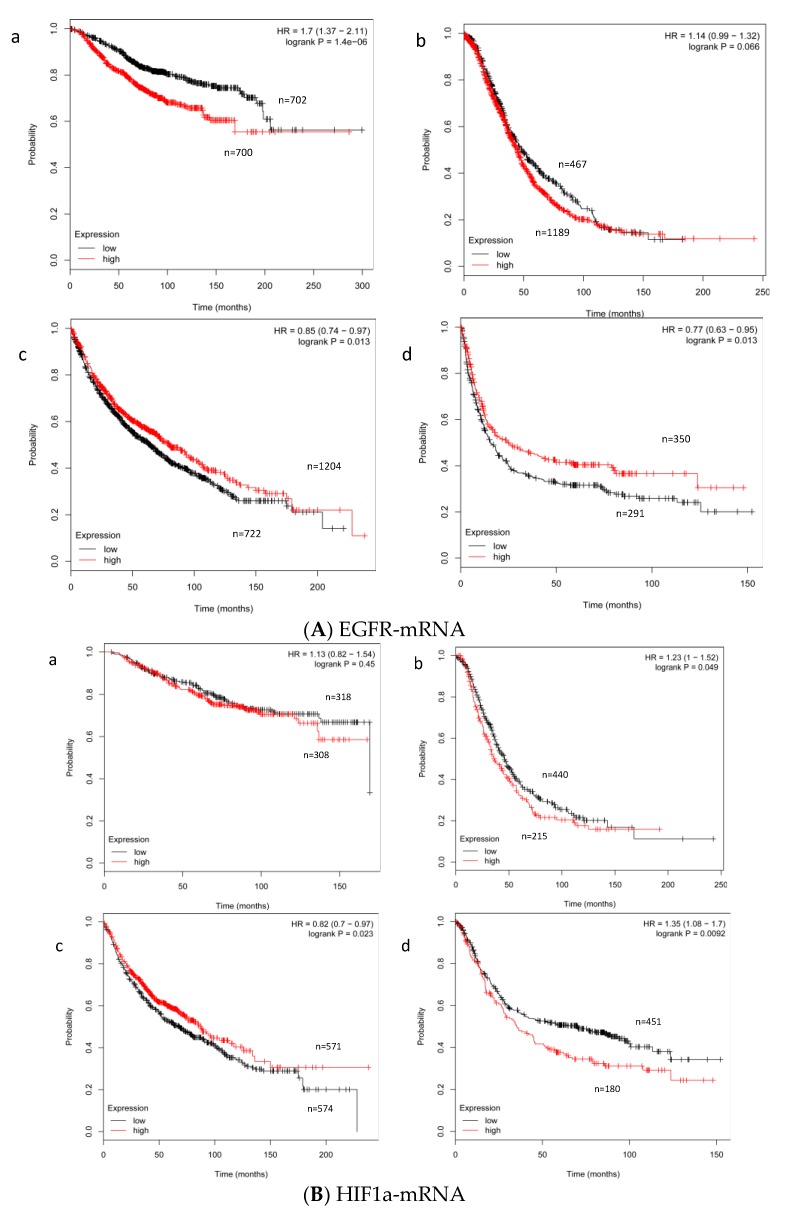
Comparative data on to the impact of EGFR (7A) and HIF-1α (7B) mRNA expression on the overall survival of breast (**a**), ovarian (**b**), lung (**c**), and gastric (**d**) cancer patients. Plots were drawn by the Kaplan-Meier Plotter algorithm (Available online: http://kmplot.com/analysis) (accessed on 22 October 2018.) usingdata of the EGFR probe 1565483-at and of the HIF-1α probe 200989-at. Abbreviations: HR—hazard ratio.

The different effect of EGFR or HIF-1α mRNA in breast, ovarian, lung, and gastric cancer (Figure 7) was also demonstrated by the Kaplan-Meier Plotter algorithm, which documents the different tissue-specific prognostic effect of both markers.

Moreover, in our study we found a significant correlation between EGFR mRNA and HIF-1α mRNA expression in the tumor tissues as well as in the normal tissues. It is known that the EGFR-pathway can affect the HIF-1α expression via the *PI3K/MAPK-AKT* pathway [3,39]. However, Secades et al. found in HNSCC-derived cells that EGF induced HIF-1α under normoxia at the protein, but not the mRNA level [26].

Wang et al. showed that hypoxia and activation of HIF prolongs the activation and the half-life of the EGF-receptor [40], and epigenetic regulation of EGFR may also play a role. Three microRNAs (miR-145, miR-21-5p, and miR-27a-3p) all activated by HIF-1α [41,42,43] can affect EGFR expression. Mir-145 downregulates EGFR mRNA and protein [44], miR-27a-5p reduces only EGFR protein but not EGFR mRNA [45], and an inhibition of miR-21-5p is associated with reduction of EGFR protein [46]. In this sense, the process from the mRNA level to protein level can be affected, for example, by non-coding (nc) RNAs such as miRNAs. MicroRNA-mediated gene/protein regulation in STS and miRNAs as potential targets in STS have been extensively studied, particularly for liposarcoma therapeutics [47,48,49]. In addition, other ncRNAs such as long non-coding RNAs may also act post-transcriptionally (e.g., in the inhibition of miRNA) or affect the activation and/or localization of proteins [50]. These data show the complex relationship between EGFR and HIF-1α, therefore, more in vivo studies concerning genetic/epigenetic regulation and protein interactions are necessary.

Studies show that treatment of cancer cells with EGFR-therapeutics (e.g., erlotinib, gefitinib, and cetuximab) down-regulated the levels of HIF-1α protein [51,52,53]. Therefore, cetuximab might be useful for adjuvant therapy of *PTEN* wild-type tumors that express HIF-1α protein [53]. Such an EGFR-specific treatment option was performed in a phase II trial for patients with synovial sarcomas [54] and could be considered for other mesenchymal tumors that express HIF-1α.

## 4. Material and Methods

### 4.1. Tissue Samples and Histopathologic Data

We examined 114 frozen tumor samples from STS patients as well as 19 frozen normal samples from adjacent non-tumor tissues (muscle) by multiplex real-time quantitative PCR analysis. The study was carried out in compliance with the Helsinki Declaration, and it was approved by the Ethics Committee of the Medical Faculty of the University Halle (Decision from the 24 January 2007). All patients gave written informed consent (Institute of Pathology, University of Halle, Germany and Department of Surgery 1, University of Leipzig, Germany). The patients’ median age was 59 years (ranging from 14 to 87 years). Fifty-seven patients (50%) died from their tumor after an average of 23 months (ranging from 2–119 months), while 57 patients (50%) were still alive after an average observation period (i.e., after primary tumor resection) of 59 months (ranging from 9–198 months).

The histopathological and clinical data were described previously [55] and are summarized in Table 1 and Table 2.

### 4.2. RNA Preparation, cDNA Synthesis and Transcript Analysis by Multiplex Quantitative Fluorescence PCR

Total RNA was prepared from 5-µm tissue slices (5 µm each). The tissue was incubated in Trizol (Thermo Fisher Scientific, Waltham, MA, USA) for 5 min at room temperature, then mixed with chloroform (AppliChem, Darmstadt, Germany) and centrifuged. The RNA was incubated with DNAse (Qiagen, Hilden, Germany) digestion. Then, the RNA was precipitated with isopropanol (AppliChem, Darmstadt, Germany) for 12 h at 4 °C and washed with ethanol solutions (96% and 70%). RNA was dissolved in RNAse-free water (Qiagen, Hilden, Germany). RNA concentrations were assessed spectrometrically. The cDNA synthesis was performed with a RevertAid First strand cDNA synthesis kit (Thermo Fisher Scientific, Waltham, MA, USA). Briefly, 1 µg RNA was used for cDNA synthesis with random hexamer primers in a Thermo-Trioblock TB1 (Biometra, Göttingen, Germany) according to the manufacturer’s instructions and as previously described in Greither et al. [56].

The mRNA levels of HIF-1α, EGFR, and the reference gene (HPRT) were quantified in a simultaneous detection assay using the Plexor multiplex qPCR-system (Promega, Heidelberg, Germany) in a Rotorgene 6000 (LTF, Wasserburg, Germany).

The Plexor multiplex qPCR assays were carried out in 15 µL of a reaction mixture comprised of 1 µL of the cDNA reaction, 0.1 µM of each specific primer, and 7.5 µL of the 2× Plexor mastermix. Cycling conditions consisted of a single activation step at 95 °C for 2 min followed by 40 cycles at 95 °C for 15 s and 60 °C for 30 s, where fluorescence measurements were taken at the end of the elongation step of each cycle. The primer pairs that were used for the detection of *EGFR, HIF-1α*, and *HPRT* were designed with the Plexor™ Primer Design Software (www.promega.com/plexorresource/) (accessed on 17 January 2008). The primer pairs that were used for detection were as follows: HIF-1α forward, 5’-CAGGACAGTACAGGATGCTTGC-3’ and HIF-1α reverse, Cy5^®^isodC-5’-GCACTGTGGTTGAGAATTCTTGG-3’; *EGFR* forward, 5’-AAAGTTAAAATTCCCGTCGCTATCAAG-3’ and *EGFR* reverse, Texas Red^®^isodC-5’-TCACGTAGGCTTCATCGAGGATTTC-3’; *HPRT* forward, HEXisodC-5’-TTGCTGACCTGCTGGATTAC-3’ and *HPRT* reverse 5’-CAGTCTGATAAAATCTACAGTCATAGG-3’. A 97-nt PCR product for HIF-1α, an 87-nt PCR product for EGFR, and an 82-nt PCR product for HPRT were amplified. For each primer pair, a melting curve analysis was applied to confirm the presence of a single amplicon. The mRNA expression of HIF-1α and EGFR was standardized to the transcript level of HPRT. The mRNA expression of HIF-1α and EGFR was calculated as copy per copy HPRT mRNA according to a standard curve generated by the use of a dilution series of the gene primer-specific amplificates. Samples were run on a RotorGene real-time-PCR cycler (LTF Labortechnik, Wasserburg, Germany) using the Rotor-Gene 6000 series Software 1.7.87.

### 4.3. Protein Isolation and ELISA Procedure

Total protein was isolated from frozen tumor tissue using a 1 ml extraction buffer (20 mM Tris, 125 mM NACL, 1% TritonX-100 ph 8.5 plus 1:100 HALT Phosphatase-Inhibitor (Thermo Scientific, Waltam, USA) and 1:100 Protease-Inhibitor (Sigma, St. Louis, MO, USA)) and incubated for 30 s on ice and homogenized via ultrasound. The protein solution was then incubated for 4 h at 4 °C on a roller mixer and centrifuged for 15 min at 15,000 rpm at 4 °C. The concentration of the protein solution was determined by the Bradford method. The protein solution was subsequently stored at −80 °C.

To detect the EGFR level in the tumor protein solution, the STAR EGFR ELISA kit from Millipore (Billerica, USA) was used according to the manufacturer’s instructions. Briefly, 100 µL diluted protein samples (1:10 in diluted buffer) were incubated for 2 h at 25 °C in a 96-well plate, then the well was washed four times with wash buffer, incubated with 100 µL detection antibody for 1 µL, washed with wash buffer, followed by an incubation with 100 µL secondary antibody for 45 min. After washing four times with washing buffer, the samples were incubated with 100 µL TMB solution for 10–45 min, the reaction was finished with 100 µL stop solution, and then a measurement by plate reader at 450 nm was performed. The concentration of EGFR- level in each sample was calculated using a standard curve. 

To determine the level of HIF-1α protein, the Milliplex ®MAP Kit (Billerica, USA) with HIF-1α MAPmates™, Phospho-AKT/PKB (SER473) MAPmates™, and Phospho PTEN(Ser380) MAPmates™ for a multiplex ELISA was applied. The kit was used as described according to the manufacturer’s instructions. Briefly, the tissue lysate was diluted 1:1 with assay buffer 2 (12.5 µL) and incubated with 25 µL of 3× beads with shaking for 2 h at room temperature (RT). Then, the solution was washed twice with 100 µL assay buffer and incubated with 25 µL detection antibodies for 1 µL at RT, followed by antibodies removal and incubation of 25 µL diluted streptavidin–phycoerythrin for 15 min at RT. After adding 25 µL amplification buffer for 15 min, the solution was removed and the beads were incubated in 150 µL assay buffer und measured using the Bio-Plex Reader (Bio-Rad Laboratories, Hercules, CA, USA).

## 5. Statistical Analysis

The Cox’s regression hazard model was used to estimate an association between HIF-1α or EGFR mRNA/protein expression and the tumor-specific survival of STS patients. The model was adjusted for the prognostic effects of covariates (tumor stage, tumor entity, tumor localization, and type of tumor resection). The interrelationship between gene expression levels was tested with the Spearman’s rank correlation (tumor tissue) or Pearson’s test for bivariate correlations (normal distribution) in the normal tissue. We tested the normal distribution of the data using the Kolmogorov-Smirnov test. For the test of correlation with T-stage, N-stage, grading, and gender of the patients, the Kruskal Wallis test was used.

Additional Kaplan-Meier survival analyses for HIF-1α or EGFR mRNA expression in breast, lung, ovarian, and gastric carcinoma samples were carried out by the Kaplan-Meier Plotter algorithm (Available online: http://kmplot.com/analysis/) (accessed on 22 October 2018) [56]. A probability (p) of <0.05 was defined as significant and the relative risk (RR) was calculated. The statistical analysis was carried out using SPSS software version 25.0 (SPSS Inc., Chicago, IL, USA).

## 6. Conclusions

For the first time, we showed that attenuated levels of EGFR mRNA or HIF-1α mRNA are significantly independent, negative prognostic markers for STS patients. In addition, there is a significant association between EGFR mRNA and HIF-1α mRNA expression. These findings can help in the understanding of the molecular pathways involved in STS development and allow for estimation of the individual prognoses of STS patients.

## Figures and Tables

**Table 1:** Histopathological, clinical, and mRNA expression data.

	*Total (n = 114)*	*Low EGFR (n = 57)*	*High EGFR (n = 57)*	*Low HIF-1α (n = 57)*	*High HIF-1α (n = 57)*
			*p* = 0.25		*p* = 0.70
Men/Women	49/65	28/29	21/36	26/31	23/34
*Tumor type*			*p* = 0.62		*p* = 0.80
liposarcoma	26	15	11	15	11
fibrosarcoma	7	2	5	3	4
UPS	27	10	17	11	16
NS	11	6	5	6	5
RMS	8	5	3	5	3
LMS	19	11	8	10	9
Other STS	16	8	8	7	9
*Tumor grade*			*p* = 0.51		*p* = 0.88
I	19	10	9	10	9
II	49	26	23	23	26
III	46	21	25	24	22
*Tumor stage*			*p* = 0.91		*p* = 0.79
I	16	7	9	8	8
II	47	26	21	24	23
II	39	16	23	20	19
IV	12	8	4	5	7
*Tumor localization*			*p* = 0.69		*p* = 0.67
extremities	73	35	38	37	36
thorax	11	7	4	7	4
head	4	2	2	2	2
abdomen	24	12	12	10	14
multiple	2	1	1	1	1
*Relapses*			***p* = 0.004**		*p* = 0.18
no	68	42	26	38	30
yes	46	15	31	19	27
*Lymph node status*			*p* = 0.44		*p* = 1.00
N0	107	52	55	54	53
N1 or higher	7	5	2	3	4
*Distant metastasis*			*p* = 0.49		*p* = 1.00
M0	105	51	54	53	52
M1	9	6	3	4	5
*Tumor resection*			*p* = 0.16		*p* = 0.84
radical (R0)	78	43	35	40	38
not radical (R1)	36	14	22	17	19
*Patients at follow-up*			*p* = 0.71		*p* = 0.13
alive	57	27	30	24	33
dead	57	30	27	33	24

*EGFR*, epidermal growth factor-receptor; *HIF-1α*, hypoxia inducible factor-1α; LMS, leiomyosarcoma; NS, neurogenic sarcoma; PS, pleomorphic sarcoma; RMS, rhabdomyosarcoma; STS, soft tissue sarcoma; SyS, synovial sarcoma; UPS, undifferentiated pleomorphic sarcoma. Bold represent the significant results

**Table 2:** Histopathological, clinical, and protein expression data.

	*Total (n = 74)*	*Low EGFR (n = 37)*	*High EGFR (n = 37)*	*Low HIF-1α (n = 37)*	*High HIF-1α (n = 37)*
			*p* = 0.64		*p* = 0.35
Men/Women	37/37	20/17	17/20	21/16	16/21
*Tumor type*			*p* = 0.04		*p* = 0.45
liposarcoma	19	14	5	12	7
fibrosarcoma	2	0	2	1	1
UPS	16	7	9	6	10
NS	5	3	2	2	3
RMS	6	5	1	3	3
LMS	14	4	10	9	5
Other STS	12	4	8	4	8
*Tumor grade*			*p* = 0.65		*p* = 0.61
I	13	9	4	8	5
II	32	13	19	15	17
III	29	15	14	14	15
*Tumor stage*			*p* = 0.94		*p* = 0.60
I	10	8	2	7	3
II	31	12	19	14	17
II	24	10	14	11	13
IV	9	7	2	5	4
*Tumor localization*			*p* = 0.22		*p* = 0.12
extremities	47	25	22	20	27
thorax	6	5	1	4	2
head	2	2	0	1	1
abdomen	17	4	13	11	6
multiple	2	1	1	1	1
*Relapses*			*p* = 0.34		*p* = 0.63
no	45	25	20	24	21
yes	29	12	17	13	16
*Lymph node status*			*p* = 0.11		*p* = 0.62
N0	70	33	37	34	36
N1 or higher	4	4	0	3	1
*Distant metastasis*			*p* = 0.43		*p* = 1.00
M0	67	32	35	33	34
M1	7	5	2	4	3
*Tumor resection*			*p* = 0.14		*p* = 1.00
radical (R0)	49	28	21	24	25
not radical (R1)	25	9	16	13	12
*Patients at follow-up*			*p* = 1.00		*p* = 0.48
alive	34	17	17	19	15
dead	40	20	20	18	22

*EGFR*, epidermal growth factor-receptor; *HIF-1α*, hypoxia inducible factor-1α; LMS, leiomyosarcoma; NS, neurogenic sarcoma; PS, pleomorphic sarcoma; RMS, rhabdomyosarcoma; STS, soft tissue sarcoma; SyS, synovial sarcoma; UPS, undifferentiated pleomorphic sarcoma. Bold represent the significant results
